# Comparative Evaluation of Packing Models for Mix Design and Performance Optimization of Ceramsite-Modified Lightweight Ultra-High-Performance Concrete

**DOI:** 10.3390/ma19112329

**Published:** 2026-06-01

**Authors:** Wanqing Zhou, Liangcheng Wang, Mengjie Jiang, Dongmei Liu, Yanzhou Peng

**Affiliations:** College of Civil Engineering and Architecture, China Three Gorges University, Yichang 443002, China; zhouwanqing@ctgu.edu.cn (W.Z.);

**Keywords:** lightweight ultra-high-performance concrete, ceramsite sand, excess paste theory, modified Andreasen model, compressible packing model, steel fiber, shrinkage behavior

## Abstract

Lightweight aggregates have a porous structure and high water absorption, which may lead to underestimation of the powder content in conventional mix design methods for lightweight ultra-high-performance concrete (LUHPC). To address this issue, this study used ceramsite sand as the lightweight aggregate and combined the excess paste theory with the particle packing method to design and evaluate ceramsite-sand-based LUHPC mixtures based on the modified Andreasen packing model (APM) and the compressible packing model (CPM). By optimizing the particle size distribution of ceramsite sand and the binder composition, a mix design method suitable for ceramsite-sand-based LUHPC was developed. The workability, apparent density, mechanical properties, elastic modulus, and shrinkage behavior of the material with different steel fiber contents were systematically investigated. The results showed that the total binder content, water-to-binder ratio, and paste volume of the mixtures designed using the two models differed only slightly. However, the aggregate skeleton formed by CPM was denser, and its skeleton packing volume was approximately 3.5% lower than that obtained using APM. At the same steel fiber content, the main mechanical properties of the CPM-designed LUHPC were generally superior to those of the APM-designed mixtures. Specifically, the 28-day cube compressive strength increased by 5.0–7.6%, the axial compressive strength by 8.8–12.2%, the axial tensile strength by 6.4–25.8%, the flexural strength by 14.1–17.2%, and the shear strength by 3.1–6.5%. The elastic modulus was also slightly higher, while the shrinkage remained consistently lower. The CPM-2.0 LUHPC mixture achieved a 28-day cube compressive strength of 124.6 MPa and an apparent density of approximately 1982 kg/m^3^, realizing a compressive strength above 120 MPa at a density below 2000 kg/m^3^. The 28-day cube compressive strength of the CPM-3.0 mixture further increased to 131.7 MPa. As the steel fiber content increased from 1.5% to 3.0%, the workability of LUHPC decreased, whereas its compressive, tensile, flexural, and shear properties generally improved, and the elastic modulus increased slightly. Steel fibers effectively restrained shrinkage deformation, but the improvement showed diminishing marginal benefits with increasing fiber content. Considering the mechanical performance, shrinkage control, and material economy, a steel fiber content of approximately 2.0% is recommended as a reference range for ceramsite-sand-based LUHPC. Overall, CPM is more suitable than APM for the mix design of ceramsite-sand-based LUHPC and can provide guidance for mix optimization and performance regulation of lightweight ultra-high-performance concrete.

## 1. Introduction

With the increasing demands on material performance in engineering construction, ultra-high-performance concrete (UHPC) has been widely applied because of its outstanding mechanical properties and durability. However, its high density and pronounced shrinkage limit its use in fields such as prefabricated construction and super-high-rise buildings. Lightweight ultra-high-performance concrete (LUHPC), in which lightweight aggregates are used to partially replace fine aggregates, combines the advantages of high strength, low density, and reduced shrinkage, and has therefore become a major research focus. The use of ceramsite as a lightweight aggregate for the preparation of LUHPC not only aligns with national strategies for energy conservation and emission reduction, but also enables the effective utilization of solid waste resources, offering clear environmental benefits.

Most existing mix design methods for LUHPC are adapted from those developed for UHPC, for which substantial research has been conducted both in China and internationally. Furnas [1] was the first to apply mathematical theory to improving particle packing density, and particle packing models subsequently became a major research focus. Long et al. [2] used the Aim and Goff models to investigate the influence of mineral admixture particle size on system compactness, whereas Hu et al. [3] and Yu et al. [4] optimized the particle gradation of UHPC using the Andreasen packing model. In terms of mechanical performance, Hasnat et al. [5] and Shi et al. [6] systematically investigated how constituent materials, particularly supplementary cementitious materials, affect the properties of UHPC, whereas Yoo et al. [7], Gao [8], and Chun and Yoo [9] examined in depth the reinforcing effects and mechanisms of steel fibers in UHPC. Research on LUHPC has also progressed steadily. Wang et al. [10] applied the densest packing theory to develop a material with a density below 1950 kg/m^3^ and a compressive strength exceeding 80 MPa. Zhang et al. [11] and Lu et al. [12] further confirmed that the type of lightweight aggregate has a significant influence on LUHPC performance. Yan [13] prepared LUHPC using hollow glass microspheres and ceramsite, achieving a density of 2031 kg/m^3^ and a compressive strength of 112.1 MPa. Regarding shrinkage behavior, Wu et al. [14], Chen et al. [15], and Gao et al. [16] reported that steel fibers can effectively reduce the shrinkage of UHPC, although this beneficial effect becomes less pronounced when the fiber content exceeds 2%. Oesterlee et al. [17] further showed that steel fibers can suppress the formation of early-age cracks by alleviating capillary shrinkage stress, dispersing local stresses, and limiting microcrack propagation.

Current mix design methods for lightweight ultra-high-performance concrete (LUHPC) mostly follow the equal-volume replacement approach used for conventional UHPC. However, they lack systematic theoretical support for the porous characteristics of lightweight aggregates, which may result in unstable performance and relatively high cost. In this study, the density conversion relationship between LUHPC and UHPC was derived based on the specific gravity relationship between lightweight aggregate concrete and normal-weight concrete. A performance target of compressive strength ≥ 120 MPa and density ≤ 2000 kg/m^3^ was proposed, and low-cost ceramsite sand was used to prepare LUHPC. This study focuses on comparing the differences and applicability of two widely used particle packing models in mix design: the modified Andreasen packing model (APM) and the compressible packing model (CPM). APM is based on a continuous particle size distribution and can flexibly fit the grading curve by adjusting the distribution modulus, making it suitable for fine-particle systems. However, its parameters strongly depend on empirical correction, and it does not consider the influence of internal pores in lightweight aggregates on packing volume. In contrast, CPM is based on gap grading and simulates the actual compaction process through stepwise filling, which better reflects the physical mechanism of aggregate packing. Nevertheless, CPM uses the mean particle size to represent particle groups, making it difficult to accurately describe the real particle size distribution. It also neglects the fact that the pores of lightweight aggregates can be filled by powders, and thus may underestimate the actual binder content required. Therefore, after applying the two models separately to optimize the aggregate and binder gradations to their theoretically densest states, this study introduced the excess paste theory and incorporated the volume of open pores in lightweight aggregates into the paste volume calculation, thereby compensating for the underestimation of powder content caused by aggregate porosity. By comparing the workability, mechanical properties, and shrinkage behavior of LUHPC designed using the two models, this study systematically reveals the differences between their design pathways and provides a basis for establishing a mix design theory suitable for high-performance concrete with porous lightweight aggregates.

## 2. Design and Preparation of LUHPC

### 2.1. Raw Materials

Lightweight ultra-high-performance concrete (LUHPC) was prepared using P·O 52.5-grade ordinary Portland cement, ultrafine fly ash, silica fume, shale ceramsite sand, a polycarboxylate-based high-performance superplasticizer, and straight copper-coated steel fibers. The cement had a specific surface area of 413 m^2^/kg, an apparent density of 3150 kg/m^3^, and a loss on ignition of 1.36%. Its 3-day and 28-day compressive strengths were 30.7 MPa and 60.4 MPa, respectively, and its initial and final setting times were 151 min and 204 min, respectively. The fly ash had a specific surface area of 1502 m^2^/kg, an apparent density of 2440 kg/m^3^, a bulk density of 1120 kg/m^3^, a residue of 16% on the 5 μm sieve, and a loss on ignition of 2.62%. The silica fume had an SiO_2_ content greater than 96%, a specific surface area of 22,453 m^2^/kg, an apparent density of 2220 kg/m^3^, an alkali content of 0.18%, a water demand ratio of 112%, and a loss on ignition of 1.48%.

Ceramsite sand was used as the lightweight aggregate. It had a particle size range of 0–3 mm, an apparent density of 1835 kg/m^3^, a bulk density of 819 kg/m^3^, a cylinder crushing strength of 6.8 MPa, and a water absorption of 16.34%. The polycarboxylate-based high-performance superplasticizer had a water-reducing rate of 35%, an air content of 3%, an initial setting time difference of 120 min, and a 28-day shrinkage ratio of 100%. The steel fibers were straight copper-coated steel fibers with a length of 13 mm, a diameter of 0.2 mm, a tensile strength of 2850 MPa, an elastic modulus of 200 GPa, and a density of 7850 kg/m^3^.

The particle size distributions of the powdered materials were determined using a Microtrac S3500 laser particle size analyzer (Microtrac, Newtown, PA, USA; measurement range: 0.02–2000 μm) based on laser diffraction, while the particle size distribution of the ceramsite sand was measured using the standard sieve-shaking method. The specific surface areas of cement and fly ash were determined using the Blaine air permeability method, and the specific surface area of silica fume was verified according to the value provided in the material testing report. The apparent densities of the powdered materials were measured using the Le Chatelier flask method, and the bulk density of fly ash was determined under the natural loose-packing condition. The apparent density, bulk density, cylinder crushing strength, and water absorption of the ceramsite sand were tested according to Lightweight Aggregates and Their Test Methods [18]. The basic performance parameters of the superplasticizer and steel fibers were obtained from material testing reports, and key indicators such as density and geometric dimensions were verified before the experiments.

### 2.2. Design of Mix Proportion

The mix design in this study consisted of three steps. First, the particle size distribution of ceramsite sand and the binder composition were optimized using the modified Andreasen packing model (APM) and the compressible packing model (CPM), respectively. Second, considering the porous and water-absorbing characteristics of ceramsite sand, the volume of open pores inside the ceramsite sand and the volume of paste coating its surface were incorporated into the conventional excess paste theory to calculate the required paste volume. Finally, the water-to-binder ratio, superplasticizer dosage, and steel fiber content range were determined through preliminary tests, and the mix proportions of each LUHPC group were obtained using the absolute volume method.

Both the Andreasen model and CPM only consider the external volume fraction of aggregates and do not account for the ability of open pores in lightweight aggregates to be filled with powders. Therefore, using packing models alone for mix design may underestimate the required powder content. To address this issue, the excess paste theory was adopted to accurately calculate the paste demand, thereby supplementing the previously underestimated powder materials and avoiding the underestimation of powder content caused by the porous nature of lightweight aggregates.

(1) Andreasen Packing Model

In this study, the modified Andreasen model, which considers both the maximum and minimum particle sizes, was adopted to design the aggregate gradation, as expressed in Equations (1) and (2).

(1) $$p^{\prime }\left(d\right)=\frac{d^{q}-d_{min}^{q}}{d_{max}^{q}-d_{min}^{q}}$$

(2) $$RSS=\sum_{i=1}^{n}{\left[U_{t}(D_{i})-U_{m}(D_{i})\right]}^{2}$$

where $p^{\prime }\left(d\right)$ is the volume fraction of aggregate particles with a particle size smaller than d; $d_{min}$ is the minimum particle size; $d_{max}$ is the maximum particle size; q is the distribution modulus, taken as 0.25; RSS represents the sum of mean squared errors; $U_{t}\left(D_{i}\right)$ is the target grading curve; and $U_{m}\left(D_{i}\right)$ is the particle size distribution curve of the solid mixture.

The proportions of the individual cementitious materials were determined by MATLAB programming. MATLAB R2021a (MathWorks, Natick, MA, USA) was used for the constrained optimization and enumeration calculations of the packing models. First, the particle size distributions of the components in the cementitious system were measured to identify the maximum and minimum particle sizes of the materials. Equation (1) was then plotted as the target curve, and the mass fractions of the individual components in the blended cementitious material were adjusted until the particle size distribution curve of the blended cementitious system showed the highest degree of agreement with the target curve. On this basis, the dosage of each material in the cementitious system was determined. The calculation method is given in Equation (2).

In the lightweight ultra-high-performance concrete system, the paste mainly serves two functions. First, it fills the voids in the aggregate packing network, including both the interparticle voids and the open pores within the lightweight aggregate. Second, it forms a coating layer of a certain thickness on the aggregate surface, thereby reducing interparticle friction and improving the flowability of the concrete. In view of the high porosity of ceramsite, the conventional excess paste theory was modified in this study.

For ordinary dense aggregates, the paste is mainly used to fill the voids between particles. For porous lightweight aggregates such as ceramsite, however, the paste can further penetrate into the open pores inside the aggregate during mixing. Therefore, to accurately reflect the actual paste demand, it is necessary to consider the influence of the internal pores of the aggregate in addition to the conventional packing voids.

According to Lightweight Aggregates and Their Test Methods [18], the conventional packing void ratio of the blended ceramsite can be calculated from the measured bulk density and apparent density, as shown in Equation (3).

(3) $$\partial =\left(1-\frac{\gamma }{\rho }\right)$$

where ∂ is the conventional packing void ratio of the blended aggregate; γ is the bulk density of the blended aggregate; and ρ is the apparent density of the blended aggregate.

For ceramsite particles, in a loosely packed aggregate system of 1 m^3^, the voids that the paste actually needs to fill should include two parts. The first is the voids between particles, whose volume fraction is equal to the conventional packing void ratio. The second is the open pores within the aggregate particles, whose volume fraction is equal to the product of the apparent volume fraction of the aggregate and the open-pore ratio of the particles themselves. Accordingly, the total volume fraction of voids to be filled in the system is defined and calculated using Equation (4).

(4) $$\phi =\partial +\left(1-\partial \right)\varphi$$

where ϕ is the total volume fraction of voids that need to be filled by paste, taking 1 m^3^ of loosely packed aggregate as the reference; and φ is the volume fraction of open pores within the ceramsite particles themselves.

To determine the amount of excess paste required to form a surface lubrication layer, the total surface area of aggregate per unit volume must be calculated. Assuming that the aggregate particles are ideal spheres, the average particle size of each size fraction *i* is $D_{i}$, and its volume fraction is $K_{i}$. Considering the irregular shape of actual ceramsite particles, a shape correction coefficient β was introduced and, according to the preliminary test results, was taken as 0.95. The theoretical surface area of aggregate per unit absolute solid volume can be calculated using Equation (5), and the actual total surface area of aggregate per cubic meter of loosely packed aggregate can then be obtained from Equation (6).

(5) $$S_{0}=\sum {(K}_{i}\times \frac{6}{D_{i}})$$

(6) $$S=S_{0}\times \left(1-\partial \right)\times \beta$$

where $S_{0}$ is the surface area of aggregate per unit absolute solid volume under the ideal spherical-particle assumption, and S is the actual total surface area of aggregate per cubic metre of packed aggregate after correction.

According to the modified excess paste theory, the total paste volume required for each cubic meter of loosely packed aggregate consists of two parts: the paste volume required to fill the total voids to be filled and the paste volume required to form a coating layer on the aggregate surface. The calculation is given in Equation (7).

(7) $$V_{p}=APT\cdot S+\phi$$

where $V_{p}$ is the total paste volume required per cubic metre of packed aggregate, and APT is the prescribed average thickness of the paste coating layer, which was taken as 7 μm in this study.

It should be noted that Equation (7) gives the relative paste demand based on 1 m^3^ of loosely packed aggregate. In the actual preparation of 1 m^3^ of LUHPC, this value should be converted according to the absolute volume method. When a paste volume of $V_{p}$ is added to 1 m^3^ of loosely packed aggregate, the paste will first fill the total voids to be filled in the system, and only the remaining portion will push the aggregate particles apart, thereby increasing the overall volume of the mixture. Therefore, the absolute total volume of the mixture can be expressed as the sum of the solid volume of the aggregate and the total paste volume.

Considering the unavoidable air content in concrete, the minimum absolute paste volume required for each cubic meter of LUHPC can be calculated using Equation (8).

(8) $$V_{pmin}=\left(\frac{V_{P}}{{1-\phi +V}_{P}}\right)\cdot \left(1-\theta \right)$$

where θ is the air content of the concrete, taken here as 1%, and $V_{pmin}$ is the minimum absolute paste volume corresponding to 1 m^3^ of LUHPC.

The calculation results based on the Andreasen packing model are as follows:

The gradation of ceramsite was calculated using Equation (1), and the results are presented in Table 1.

The particle size and density parameters of the ceramsite sand used for the calculation are listed in Table 2.

During the optimization of the ceramsite sand gradation, the mass fractions of three ceramsite sand size ranges, namely 0.3–0.6 mm, 0.6–1.18 mm, and 1.18–2.36 mm, were used as the optimization variables. First, the target cumulative passing percentage was calculated using the modified Andreasen packing model (APM), and the target gradation curve was obtained. Then, based on the sieving results, apparent density, and bulk density of each ceramsite sand fraction, different combinations of mass fractions were converted into volume fraction combinations, and the actual particle size distribution curve of the blended ceramsite sand was calculated. The optimization objective was to minimize the residual sum of squares (RSS) between the actual gradation curve and the target gradation curve. The constraints were that the sum of the mass fractions of the three ceramsite sand fractions was equal to 1, and each mass fraction was greater than Zero. Constrained optimization using MATLAB R2021a (MathWorks, Natick, MA, USA) showed that, when RSS reached its minimum value, the mass ratio of the 0.3–0.6 mm, 0.6–1.18 mm, and 1.18–2.36 mm ceramsite sand fractions was determined to be 0.32:0.33:0.35.

**Figure 1:**
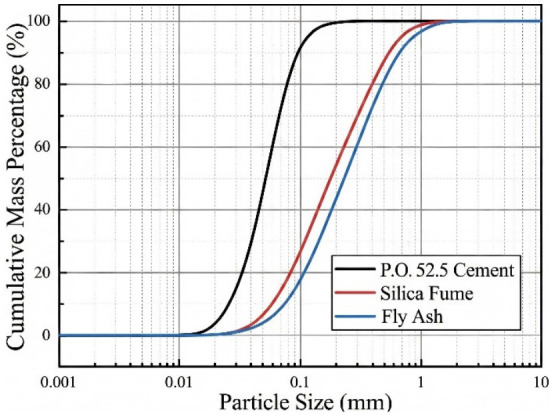
Particle Size Distribution of Cementitious Materials.

The binder composition was determined using the same optimization approach. First, the particle size distributions of cement, fly ash, and silica fume were measured, and their mass fractions were used as the optimization variables. After the mass fractions were converted into volume fractions according to the density of each material, the particle size distribution curve of the composite binder system was obtained by superposition. The objective function was to minimize the RSS between this curve and the Andreasen target curve, with the constraint that the sum of the mass fractions of cement, fly ash, and silica fume was equal to 1. The calculated mass ratio of cement, fly ash, and silica fume was 0.64:0.21:0.15. Figure 1 shows the particle size distributions of cement, fly ash, silica fume, and the optimized composite binder system. This figure further illustrates the binder gradation optimization process, in which the mass proportions of cement, fly ash, and silica fume were adjusted to make the particle gradation of the composite binder system as close as possible to the Andreasen target gradation curve. As shown in Figure 1, the composite binder system exhibited a relatively continuous particle distribution across different particle size ranges, indicating a favorable particle-filling effect among cement, fly ash, and silica fume. This provides a basis for subsequent paste densification and performance improvement of LUHPC.

If the paste demand is estimated only from the voids between particles, without considering the open pores inside the ceramsite and the coating layer on the aggregate surface, the calculated paste volume required per cubic meter of concrete is relatively small, and the corresponding mass ratio of cementitious materials to aggregate is about 1.3:1. After the modified excess paste theory is taken into account, with both the internal open pores of the ceramsite and the average paste coating layer incorporated into the calculation, the theoretically obtained mass ratio of cementitious materials to aggregate is about 1.7:1. These results indicate that the modified excess paste theory can more realistically reflect the actual distribution state of paste in a lightweight ceramsite system, which helps to ensure the flowability of the fresh mixture, improve aggregate lubrication, and avoid mix proportion deviations caused by insufficient paste.

(2) Compressible Packing Model

A key feature of the CPM compaction model is its explicit distinction between virtual compaction density and actual compaction density. To describe the relationship between these densities during the compaction process, the model introduces a compaction index, K, which characterizes the correlation between the virtual compaction density $\gamma_{i}$ and the actual compaction density $\alpha_{t}$ under different compaction conditions.

The governing equation of the model is given by:

(9) $$\gamma_{i}=\frac{\beta_{i}}{1-\sum_{j=1}^{i=1}\left[1-\beta_{i}+b_{ij}\beta_{i}\left(1-\frac{1}{\beta_{j}}\right)\right]y_{j}-\sum_{j=i+1}^{n}\left(1-a_{ij}\beta_{j}\right)y_{j}}$$

(10) $$a_{ij}=\sqrt{1-{\left.\left(1-\frac{d_{j}}{d_{i}}\right.\right)}^{1.02}}\;\left(j=i+1,\ldots ,n\right)$$

(11) $$b_{ij}=1-{\left.\left(1-\frac{d_{j}}{d_{i}}\right.\right)}^{1.50}\;\left(j=1,\ldots ,i-1\right)$$

(12) $$K=\sum_{i=1}^{n}K_{i}=\sum_{i=1}^{n}\frac{y_{i}/\beta_{i}}{1/\alpha_{t}-1/\gamma_{i}}$$

where $\gamma_{i}$ is the virtual bulk density of particles in size class i; $\beta_{i}$ is the residual bulk density of particles in size class i; $d_{i}$ and $d_{j}$ are the particle diameters of size classes i and j, respectively; $a_{ij}$ is the loosening-effect coefficient; $b_{ij}$ is the wall-effect coefficient; $y_{i}$ is the volume fraction of particles in size class i; K is the compaction index; and $\alpha_{t}$ is the actual bulk density.

The results of the CPM bulk density model are summarized below.

In the CPM optimization process, the volume fractions of the individual components were used as variables, and the maximum actual packing density of the blended system was adopted as the optimization criterion. For the ceramsite sand system, the three ceramsite sand fractions of 0.3–0.6 mm, 0.6–1.18 mm, and 1.18–2.36 mm were treated as discrete particle classes. The residual packing density was calculated based on the mean particle size, apparent density, and measured bulk density of each particle class, while the loosening effect and wall effect among particles of different size classes were also considered. The volume fraction combinations of the three ceramsite sand fractions were then varied, and the actual packing density under different combinations was calculated. The constraint was that the sum of the volume fractions of the three ceramsite sand fractions was equal to 1. In this study, the volume fraction combinations of the three ceramsite sand fractions were enumerated using a volume fraction increment of 0.1, and the maximum actual packing density was used as the optimization criterion. The final mass ratio of the 0.3–0.6 mm, 0.6–1.18 mm, and 1.18–2.36 mm ceramsite sand fractions was determined to be 0.54:0.20:0.26.

The CPM optimization method for the binder system was the same as that used for the ceramsite sand system. The volume fractions of cement, fly ash, and silica fume were used as variables, with the constraint that the sum of their volume fractions was equal to 1. Enumeration was performed using a volume fraction increment of 0.1. The actual packing density of the composite binder system under different combinations was calculated, and the maximum actual packing density was adopted as the optimization criterion. The calculation results showed that the binder system reached the optimal packing state when the mass ratio of cement, fly ash, and silica fume was 0.62:0.18:0.20.

(3) LUHPC Mix Proportion

To determine the appropriate water-to-binder ratio and superplasticizer dosage, preliminary tests were conducted based on the initial mix proportions calculated using APM. The preliminary tests examined the effects of water-to-binder ratios of 0.14–0.20 and superplasticizer dosages of 0.02–0.06 on the casting condition and flowability of the mixture. The results showed that, at a relatively low water-to-binder ratio, increasing the superplasticizer dosage had only a limited effect on improving workability. When the water-to-binder ratio was 0.18 and the superplasticizer dosage was 2.5% of the binder mass, the mixture still exhibited good casting condition and flowability under a relatively large mixing volume. Therefore, to ensure comparability between the mixtures designed using the two packing models, a water-to-binder ratio of 0.18 and a superplasticizer dosage of 0.025 were used in all subsequent tests.

On this basis, the steel fiber volume fraction was adjusted to 1.5%, 2.0%, and 3.0%, respectively, to compare the performance differences between the mixtures designed using the two packing models and to analyze the effects of steel fiber content on the workability, mechanical properties, and shrinkage behavior of LUHPC. The experimental mix proportions are shown in Table 3.

## 3. Experimental Methods

The slump and slump flow of LUHPC were measured in accordance with the Standard for Test Methods of Ultra-High Performance Concrete [19].

The apparent density was determined following the same standard. For each mixture, three specimens with dimensions of 100 mm × 100 mm × 100 mm were prepared, and the average value was reported as the final result.

As no unified standard specifically applicable to LUHPC has yet been formally established, the mechanical properties were evaluated with reference to the Standard for Test Methods of Basic Properties of Ultra-High Performance Concrete [19] and the Specification for Test Methods of Fibre Reinforced Concrete [20].

The mechanical property tests were conducted using a WAW-Y1000C microcomputer-controlled electro-hydraulic servo universal testing machine equipped with the corresponding fixtures.

Cube compressive strength was measured using 100 mm × 100 mm × 100 mm specimens. Axial compressive strength was determined using prismatic specimens of 100 mm × 100 mm × 300 mm. Axial tensile strength was evaluated using dog-bone specimens with an overall length of 368 mm × 100 mm and a central tensile section of 100 mm × 50 mm × 50 mm. Flexural strength was measured using prismatic specimens of 100 mm × 100 mm × 400 mm. Shear strength tests were conducted on 100 mm × 100 mm × 300 mm prismatic specimens. The cube compressive, axial compressive, axial tensile, flexural, shear, and elastic modulus tests were conducted using a WAW-Y1000C microcomputer-controlled electro-hydraulic servo universal testing machine.

For each test type, six groups were prepared corresponding to steel fiber volume fractions of 1.5%, 2.0%, and 3.0% under two mix design methods. Six specimens per group were tested for cube compressive strength, while three specimens per group were used for the remaining mechanical tests.

The static elastic modulus was determined in accordance with the static compression method specified in the Standard for Test Methods of Ultra-High Performance Concrete [19]. Prismatic specimens measuring 100 mm × 100 mm × 300 mm were used. For each mix design, three specimens were prepared at steel fiber volume fractions of 1.5%, 2.0%, and 3.0%, resulting in six groups in total.

The drying shrinkage test was conducted following the Dry Shrinkage Test Method for Cement Mortar [21]. For each mix, three specimens with dimensions of 25 mm × 25 mm × 280 mm were prepared.

All tests were conducted using the number of specimens specified in the corresponding standards, and the arithmetic mean of the test results was used as the representative value. In this study, the workability, apparent density, mechanical properties, elastic modulus, and shrinkage behavior of LUHPC with different packing models and steel fiber contents were compared and analyzed mainly based on the average values, relative change rates, and trends in the curves. For the mechanical property and elastic modulus results obtained from repeated specimens, standard deviations were calculated and shown as error bars in the corresponding figures.

Among these tests, cube compressive strength was used as the main indicator for evaluating the strength grade of LUHPC and the effectiveness of the mix design. Considering that local pores in lightweight aggregates, specimen compaction quality, and steel fiber distribution may affect the cube compressive strength test results, six specimens were prepared for each group in the cube compressive strength test to reduce the influence of experimental scatter on strength grade determination. The axial compressive, axial tensile, flexural, shear, and elastic modulus tests were mainly used to compare the performance variation among different mixtures. Therefore, according to the corresponding test methods and conventional experimental arrangements, three specimens were prepared for each group, and the average value was taken as the representative value.

## 4. Results and Analysis

This chapter compares and analyzes the workability, apparent density, mechanical properties, elastic modulus, and shrinkage behavior of different mixtures based on the average values, relative change rates, and curve trends obtained from the experimental results. The observed variations are further explained in terms of mix composition, particle packing state, steel fiber bridging effect, and shrinkage restraint mechanism.

### 4.1. Workability of LUHPC

**Figure 2:**
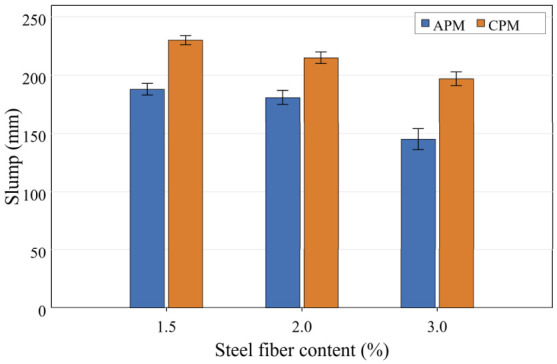
Slump Test Results.

Figure 2 shows the slump test results of LUHPC with different steel fiber contents.

As shown in Figure 2, the slump values of LUHPC designed using the Andreasen packing model were 188 mm, 181 mm, and 145 mm at steel fiber contents of 1.5%, 2.0%, and 3.0%, respectively. In comparison, the slump values of LUHPC designed using the CPM packing model were 230 mm, 215 mm, and 197 mm at the corresponding fiber contents. These results indicate that the slump of LUHPC designed using both models decreased with increasing steel fiber content. This can be mainly attributed to the increased overlap and interlocking among fibers at higher fiber contents, which intensified the internal structural blocking effect of the mixture. Meanwhile, more paste was required to coat the increased fiber surface area, thereby increasing the paste demand of the system. Consequently, the internal frictional resistance of the mixture increased, and its flowability decreased.

Further comparison shows that the LUHPC designed using CPM exhibited better overall workability than that designed using APM. Based on the mix proportion parameters discussed above, the total binder content, water-to-binder ratio, and paste volume of the mixtures designed using the two models differed only slightly. However, CPM formed a denser aggregate skeleton, and its skeleton packing volume was approximately 3.5% lower than that obtained using APM. This indicates that, under the same total paste volume, the CPM-designed mixture required less paste to fill the voids in the aggregate skeleton. As a result, more paste was available to coat and lubricate the surfaces of particles and steel fibers, reducing the internal frictional resistance of the mixture. In addition, the CPM-designed mixture contained a higher proportion of fine particles, which improved the compatibility between the particle gradation and the steel fiber network and weakened the blocking effect of coarse particles during flow. Therefore, the CPM-designed LUHPC exhibited higher macroscopic flowability and better passing ability.

### 4.2. Apparent Density of LUHPC

**Figure 3:**
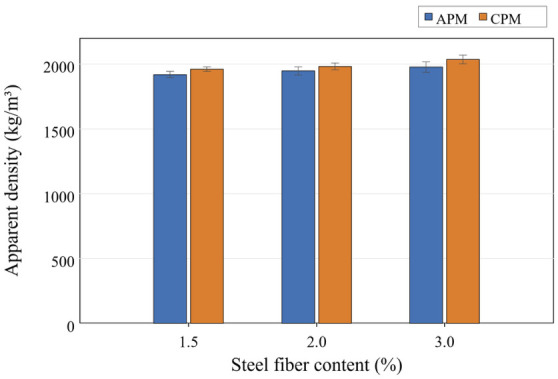
Apparent Density Test Results.

Figure 3 shows the apparent density test results of LUHPC with different steel fiber contents.

Figure 3 shows the apparent density results of the mixtures. When the steel fiber content increased from 1.5% to 3.0%, the apparent densities of the LUHPC designed using APM were 1919 kg/m^3^, 1948 kg/m^3^, and 1977 kg/m^3^, respectively. In comparison, the apparent densities of the LUHPC designed using CPM were 1961 kg/m^3^, 1982 kg/m^3^, and 2035 kg/m^3^, respectively. These results indicate that the apparent density of LUHPC designed using both models increased with increasing steel fiber content. At the same fiber content, the apparent density of the CPM-designed LUHPC was consistently higher than that of the APM-designed LUHPC.

This phenomenon can be mainly attributed to two factors. First, the density of steel fibers (7850 kg/m^3^) is much higher than that of ceramsite sand and the binder materials. Therefore, as the steel fiber content increased, the proportion of high-density components per unit volume of concrete increased, leading to a gradual increase in the apparent density of LUHPC. Second, the particle gradation optimized by CPM reduced the packing volume of the aggregate skeleton by approximately 3.5% compared with APM, thereby reducing internal pores and defects and producing a denser overall structure. As a result, the apparent density of the CPM-designed LUHPC was generally higher than that of the APM-designed LUHPC.

### 4.3. Basic Mechanical Properties of LUHPC

According to Table 3 and the raw material parameters presented in Section 2.1, the total binder contents of the APM- and CPM-designed mixtures were 995 kg/m^3^ and 1002 kg/m^3^, respectively. The corresponding water contents were 179 kg/m^3^ and 180 kg/m^3^, and the water-to-binder ratios were both approximately 0.180. The binder-to-aggregate mass ratios were also approximately 1.70:1, indicating that the LUHPC mixtures designed using the two models were essentially comparable in terms of binder phase content. Based on the actual densities of the raw materials, the paste volumes of the two mixtures were approximately 0.558 m^3^/m^3^ and 0.565 m^3^/m^3^, respectively, with a difference of approximately 0.007 m^3^/m^3^, indicating only a minor variation. Further estimation using the packing density parameters of each aggregate size fraction showed that the aggregate skeleton packing volumes of the APM and CPM mixtures were approximately 0.949 m^3^/m^3^ and 0.915 m^3^/m^3^, respectively. The latter was approximately 3.5% lower than the former, suggesting that the aggregate system designed using CPM had a denser skeleton packing state. Therefore, the subsequent differences in mechanical properties cannot be simply attributed to variations in binder content or paste volume. Instead, they should be analyzed mainly in terms of the compactness of the particle skeleton, internal pores and defects, and the synergistic interaction between steel fibers and the matrix.

#### 4.3.1. Cube Compressive Strength

**Figure 4:**
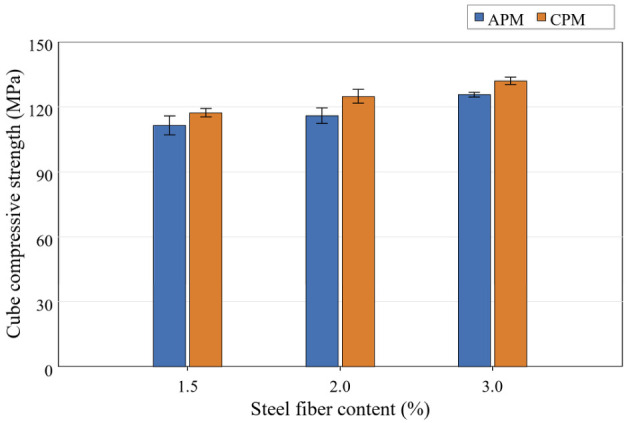
Cube Compressive Strength of LUHPC.

Figure 4 shows the cube compressive strength of LUHPC with different steel fiber contents.

Figure 4 shows the variation in the cube compressive strength of LUHPC with steel fiber volume fraction. For the APM-designed mixtures, the 28-day cube compressive strengths were 111.3 MPa, 115.7 MPa, and 125.4 MPa at steel fiber contents of 1.5%, 2.0%, and 3.0%, respectively. For the CPM-designed mixtures, the corresponding values were 117.1 MPa, 124.6 MPa, and 131.7 MPa, respectively. The cube compressive strength of LUHPC designed using both models increased with increasing steel fiber content, indicating that the fiber-bridging effect of steel fibers and their ability to restrain microcrack propagation contributed to improving the integrity and load-bearing capacity of the material. At the same steel fiber content, the 28-day cube compressive strength of the CPM-designed mixtures was higher than that of the APM-designed mixtures, with increases of 5.1%, 7.6%, and 5.0%, respectively. The relatively consistent improvement at different fiber contents indicates that the particle gradation optimized by CPM is beneficial for enhancing the compressive performance of ceramsite-sand-based LUHPC.

Because the two groups of mixtures differed only slightly in total binder content, water-to-binder ratio, and paste volume, the strength difference can be more reasonably attributed to the denser aggregate skeleton formed by the CPM-optimized particle gradation. This denser skeleton reduced internal pores and local weak zones and improved the stress coordination among the aggregate, paste, and steel fiber phases. Consequently, the load transfer efficiency was enhanced, resulting in higher cube compressive strength.

Compared with existing studies on LUHPC, the ceramsite-sand-based LUHPC prepared in this study showed certain advantages in achieving both lightweight and high-strength performance. Wang et al. [10] prepared lightweight ultra-high-performance concrete with a density lower than 1950 kg/m^3^ and a compressive strength higher than 80 MPa using the densest packing theory. Yan [13] prepared LUHPC with a density of 2031 kg/m^3^ and a compressive strength of 112.1 MPa using hollow glass microspheres and ceramsite sand. In comparison, the CPM-2.0 LUHPC mixture in this study had an apparent density of approximately 1982 kg/m^3^ and a 28-day cube compressive strength of 124.6 MPa, achieving a compressive strength above 120 MPa at a density below 2000 kg/m^3^. Zhang et al. [11] and Lu et al. [12] reported that the type of lightweight aggregate and its interfacial characteristics affect the performance of LUHPC. The results of this study further demonstrate that combining the excess paste theory with CPM can improve the particle packing state and interfacial compactness of ceramsite-sand-based LUHPC, thereby enabling a balance between lightweight design and strength enhancement.

The concrete strength grade was determined according to the standard value of cube compressive strength, and the calculation formula is as follows:

(13) $$f_{cu,k}=(1-1.645d_{c})f_{cu,m}$$

where $f_{cu,k}$ is the characteristic cube compressive strength; $f_{cu,m}$ is the mean cube compressive strength; and $d_{c}$ is the coefficient of variation, defined as the ratio of the standard deviation of the measured cube compressive strengths to the corresponding mean value.

The characteristic compressive strength of LUHPC was calculated accordingly, and the corresponding strength grade was determined and designated as LC. The results are summarized in Table 4.

#### 4.3.2. Axial Compressive Strength

**Figure 5:**
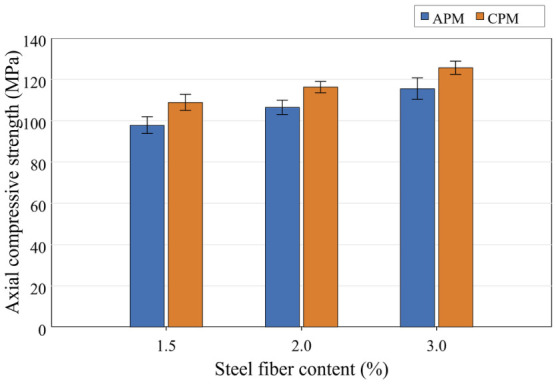
Axial Compressive Strength of LUHPC.

Figure 5 shows the axial compressive strength of LUHPC with different steel fiber contents.

The axial compressive strength of LUHPC increased with increasing steel fiber content. As shown in Figure 5, when the steel fiber volume fractions were 1.5%, 2.0%, and 3.0%, the axial compressive strengths of the CPM-designed mixtures were 109.7 MPa, 116.1 MPa, and 125.5 MPa, respectively, which were 12.2%, 9.2%, and 8.8% higher than those of the APM-designed mixtures. These results indicate that the CPM-designed mixtures exhibited higher axial compressive load-bearing capacity at different fiber contents.

Because the two groups of mixtures were similar in total binder content, water-to-binder ratio, and paste volume, the difference in axial compressive strength mainly resulted from differences in aggregate skeleton structure and internal compactness. The denser particle skeleton formed by CPM helped reduce pore defects and weak interfaces, thereby improving the continuity and uniformity of the matrix. Meanwhile, a reasonable skeleton structure also facilitated the uniform dispersion and effective anchorage of steel fibers in the matrix, allowing the fibers to more fully restrain the initiation and propagation of microcracks. Consequently, the CPM-designed mixtures exhibited higher axial compressive strength.

#### 4.3.3. Axial Tensile Strength

**Figure 6:**
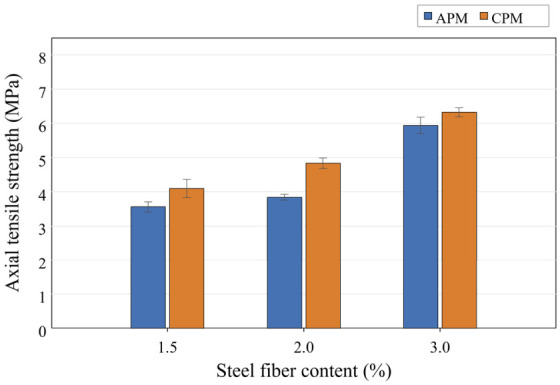
Axial Tensile Strength of LUHPC.

Figure 6 shows the axial tensile strength of LUHPC with different steel fiber contents.

Figure 6 shows that the axial tensile strength of LUHPC designed using both models increased with increasing steel fiber content. For the APM-designed mixtures, when the steel fiber content increased from 1.5% to 3.0%, the axial tensile strength increased from 3.56 MPa to 5.94 MPa. For the CPM-designed mixtures, it increased from 4.10 MPa to 6.32 MPa. These results indicate that the incorporation of steel fibers improved the axial tensile strength of LUHPC, and that the CPM-designed mixtures generally exhibited higher tensile strength than the APM-designed mixtures at the same fiber content. Specifically, at steel fiber contents of 1.5%, 2.0%, and 3.0%, the axial tensile strengths of the CPM-designed mixtures were 15.2%, 25.8%, and 6.4% higher than those of the APM-designed mixtures, respectively.

Axial tensile performance is more sensitive to internal defects and cracks; therefore, its variation more clearly reflects the differences in the synergistic effect between the particle skeleton and steel fibers. The more uniform and denser skeleton structure formed by CPM helped reduce local weak zones and stress concentration, allowing the fiber-bridging and energy-dissipation effects of steel fibers to be more fully activated. This improved the tensile load-bearing capacity and crack resistance of the material. At steel fiber contents of 1.5%, 2.0%, and 3.0%, the axial tensile strengths of the CPM-designed mixtures were 15.2%, 25.8%, and 6.4% higher than those of the APM-designed mixtures, respectively, with the greatest improvement observed at 2.0%. This indicates a favorable synergistic effect between an appropriate amount of steel fibers and the dense particle skeleton formed by CPM. When the fiber content further increased to 3.0%, the fiber quantity effect became more pronounced in both mixtures. Meanwhile, the higher fiber content may have increased fiber overlap and local interfacial defects, thereby reducing the relative contribution of the CPM skeleton advantage. As a result, the difference between the two mixtures became narrower.

#### 4.3.4. Flexural Strength

**Figure 7:**
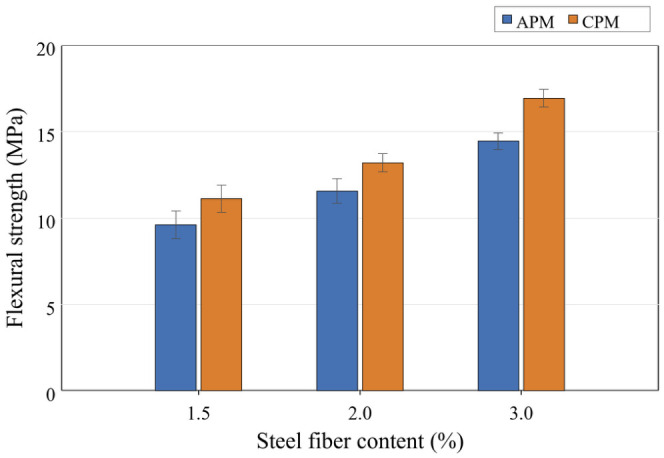
Flexural Strength of LUHPC.

Figure 7 shows the flexural strength of LUHPC with different steel fiber contents.

Figure 7 shows that the flexural strength of LUHPC increased continuously with increasing steel fiber content. For the APM-designed mixtures, the flexural strengths were 9.61 MPa, 11.56 MPa, and 14.44 MPa at steel fiber volume fractions of 1.5%, 2.0%, and 3.0%, respectively. At the same fiber contents, the flexural strengths of the CPM-designed mixtures were 14.7%, 14.1%, and 17.2% higher than those of the APM-designed mixtures, respectively.

These results indicate that the CPM-optimized particle gradation helped reduce initial defects in the flexural tensile zone and improved the anchorage and pull-out energy dissipation of steel fibers in the matrix. Flexural failure is closely related to crack propagation after matrix cracking, steel fiber bridging and pull-out, and interfacial energy dissipation. Since the two groups of mixtures differed only slightly in material proportions, the higher flexural strength of the CPM-designed mixtures can mainly be attributed to two factors. First, their aggregate skeleton was denser and more uniform, resulting in fewer initial defects and lower local stress concentration in the flexural tensile zone. Second, the reasonable particle gradation provided better anchorage conditions for steel fibers in the matrix, allowing more effective bridging and energy dissipation during crack propagation. Consequently, the CPM-designed mixtures exhibited stronger crack resistance and load-bearing capacity.

#### 4.3.5. Shear Strength

**Figure 8:**
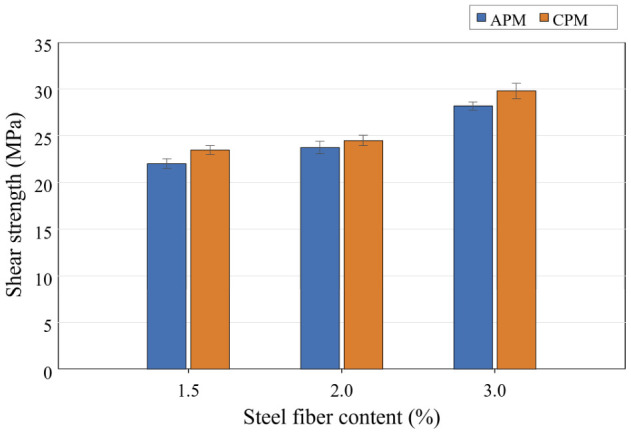
Shear Strength of LUHPC.

Figure 8 shows the shear strength results of LUHPC with different steel fiber contents.

Figure 8 indicates that the shear strength of LUHPC followed a trend similar to that of tensile and flexural properties, increasing with increasing steel fiber content. For the APM-designed mixtures, the shear strengths were 22.0 MPa, 23.72 MPa, and 28.15 MPa at steel fiber contents of 1.5%, 2.0%, and 3.0%, respectively. The corresponding shear strengths of the CPM-designed mixtures were 6.5%, 3.1%, and 5.7% higher, respectively. This suggests that the dense particle skeleton formed by CPM helps improve the resistance of LUHPC to shear failure.

Shear performance depends not only on matrix strength but also on crack surface roughness, aggregate interlock, and the crack-bridging effect of steel fibers. Under the condition that the material contents of the two groups were generally comparable, the denser particle packing skeleton formed by CPM provided a continuous and stable load transfer path. This enhanced the interlocking and bridging effects during crack propagation, thereby improving the resistance of the material to shear failure.

**Figure 9:**
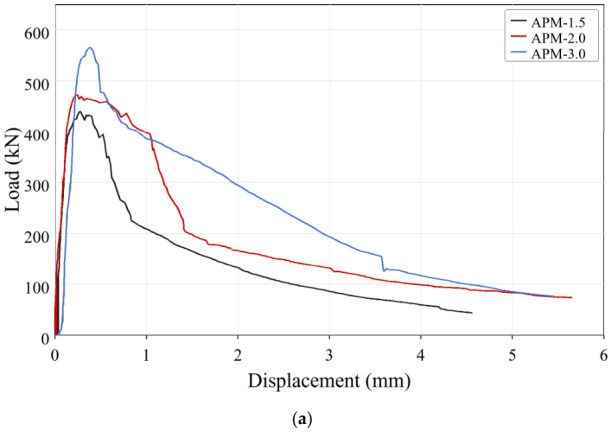

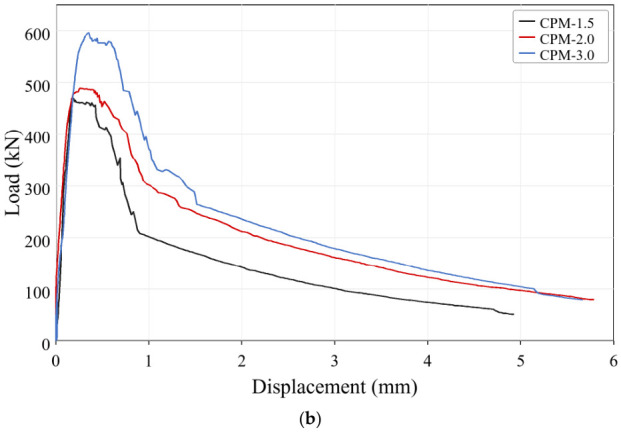
(**a**) Shear load–displacement curves of the APM group. (**b**) Shear load–displacement curves of the CPM group.

The shear load–displacement curves obtained from the tests are shown in Figure 9.

As shown by the shear load–displacement curves, the load–displacement relationship increased almost linearly before cracking, indicating that the specimens remained in an elastic stress state at this stage. After cracking, as the shear deformation increased, the load continued to increase but at a gradually reduced rate. This indicates that the steel fibers crossing the shear cracks began to carry part of the shear force through their bridging effect, and the shear transfer mechanism of the specimens changed. After the specimens reached the peak load, the load-bearing capacity decreased rapidly. This was caused by the combined effects of crack propagation along the shear failure plane, accumulated matrix damage, and progressive slip and pull-out of steel fibers. As specimen deformation continued to increase, the descending branch of the curve became more gradual, indicating that the specimens still maintained a certain residual load-bearing capacity in the post-peak stage.

Compared with the APM group, the post-peak descending branches of the CPM group were relatively more gradual, indicating better shear toughness. This suggests that the denser internal skeleton formed by the CPM-designed mixtures was beneficial for sustaining the bridging and energy-dissipation effects in the post-peak stage. This trend is generally consistent with previous studies on steel-fiber-reinforced UHPC. Yoo et al. [7] showed that increasing steel fiber content can improve the mechanical and fracture properties of UHPC. Gao [8] pointed out that the steel fiber volume fraction has an important influence on the mechanical properties of UHPC. Chun et al. [9] further demonstrated that steel fibers can improve the tensile and post-cracking load-bearing performance of UHPC by bridging cracks and enhancing fiber pull-out energy dissipation. In this study, as the steel fiber content increased from 1.5% to 3.0%, both the shear strength and post-peak residual load-bearing capacity of LUHPC increased, indicating that steel fibers can also provide crack-bridging and energy-dissipation effects in ceramsite-sand-based LUHPC. Meanwhile, the relatively gradual post-peak descending branch of the CPM-designed mixtures indicates that a denser particle skeleton helps sustain the fiber-bridging effect during shear failure.

#### 4.3.6. Modulus of Elasticity

**Figure 10:**
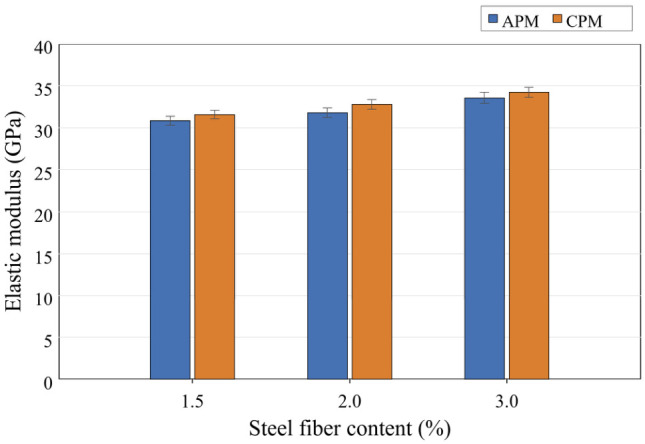
Elastic Modulus of LUHPC.

Figure 10 shows the elastic modulus of LUHPC with different steel fiber contents.

As shown in Figure 10, the elastic modulus of LUHPC increased slightly with increasing steel fiber content. For the APM-designed mixtures, the elastic moduli were 30.84 GPa, 31.79 GPa, and 33.55 GPa at steel fiber volume fractions of 1.5%, 2.0%, and 3.0%, respectively. At the same fiber contents, the elastic moduli of the CPM-designed mixtures were 2.3%, 3.1%, and 1.9% higher, respectively.

Compared with the strength indices, the difference in the elastic modulus of LUHPC between the two mixture designs was relatively small. As the steel fiber content increased, the elastic modulus of both mixtures showed a slight upward trend. At the same fiber content, the elastic modulus of the CPM-designed mixtures was slightly higher than that of the APM-designed mixtures, but the improvement was limited. Therefore, the elastic modulus is more suitable as an auxiliary indicator for evaluating changes in material stiffness, rather than as the main basis for judging the superiority of the two models.

The effect of steel fibers on improving the elastic modulus was much smaller than that on the strength indices. This is because the elastic modulus mainly reflects the overall deformation stiffness of the material at the small-strain stage and is more sensitive to matrix continuity and internal pores, while its response to fiber bridging and energy dissipation is relatively limited. The slightly higher elastic modulus of the CPM-designed mixtures indicates that their particle packing structure was denser and their matrix was more uniform, providing a more stable load transfer path in the elastic stage. However, because the two models differed only slightly in total binder content and paste volume, and because the elastic modulus was more strongly governed by matrix stiffness, the overall difference between the two groups remained limited.

### 4.4. Shrinkage Properties of LUHPC

**Figure 11:**
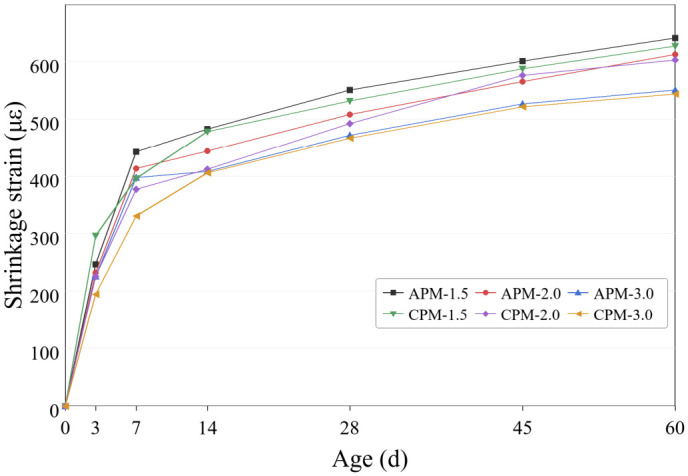
Shrinkage Deformation Curves of LUHPC with Different Fiber Contents.

Figure 11 shows the shrinkage deformation curves of LUHPC with different fiber contents.

The LUHPC mixtures designed using the Andreasen and CPM packing models exhibited the same general trend: shrinkage deformation developed rapidly from 0 to 7 days, whereas the shrinkage growth rate decreased markedly from 7 to 60 days.

For the APM-designed mixtures, when the fiber content increased from 1.5% to 3.0%, the 7-day shrinkage decreased from 442 με to 397.3 με, representing a reduction of 10.2%. The 60-day shrinkage decreased from 641 με to 551 με, corresponding to a reduction of 14.1%. Further analysis showed that the reduction in 60-day shrinkage was more pronounced when the fiber content increased from 1.5% to 2.0%, whereas the reduction became much smaller when the fiber content further increased from 2.0% to 3.0%. A similar trend was observed for the CPM-designed mixtures. The 7-day shrinkage decreased from 396 με to 330.7 με, representing a reduction of 16.5%, and the 60-day shrinkage decreased from 627.3 με to 544 με, corresponding to a reduction of 13.3%. These results indicate that increasing the steel fiber content effectively reduced the shrinkage strain of ceramsite-sand-based LUHPC.

The shrinkage-restraining mechanism of steel fibers can be explained as follows. The incorporation of steel fibers can disperse capillary shrinkage stress and relieve stress concentration. The randomly distributed fibers can also generate a hoop-confinement effect that limits deformation, while their high elastic modulus provides skeleton support and reduces early-age shrinkage. However, when the fiber content is excessively high, the increased number of interfacial transition zones and the development of additional capillary pores in LUHPC may weaken the shrinkage-restraining effect, resulting in diminishing marginal benefits. At later ages, the shrinkage of the CPM-designed mixtures was generally slightly lower than that of the corresponding APM-designed mixtures. This can be attributed to the denser particle packing, lower internal porosity, and reduced capillary negative pressure in the CPM-designed mixtures, which provided better shrinkage control. Considering mechanical performance, shrinkage control, and economy, a steel fiber content of approximately 2.0% is recommended. The findings of this study regarding the shrinkage-restraining effect of steel fibers and its diminishing marginal benefit are generally consistent with previous UHPC shrinkage studies. Wu et al. [14], Chen et al. [15], and Gao et al. [16] showed that steel fibers can effectively improve the shrinkage performance of UHPC, but their beneficial effect weakens once the fiber content exceeds a certain range. Oesterlee et al. [17] further reported that steel fibers can suppress early crack formation by reducing capillary shrinkage stress, dispersing local stress, and limiting microcrack propagation. The present results indicate that this diminishing marginal benefit also exists in ceramsite-sand-based LUHPC systems.

## 5. Conclusions

(1) To address the problem that powder content is easily underestimated in conventional mix design because of the porous nature of lightweight aggregates, this study combined the excess paste theory with particle packing models and established a mix design method suitable for ceramsite-sand-based LUHPC. Based on APM and CPM, the gradations of ceramsite sand with particle size ranges of 0.3–0.6 mm, 0.6–1.18 mm, and 1.18–2.36 mm were determined. The corresponding mass ratios were 0.32:0.33:0.35 and 0.54:0.20:0.26, respectively. The mass ratios of cement, fly ash, and silica fume were 0.64:0.21:0.15 and 0.62:0.18:0.20, respectively. The CPM-2.0 LUHPC mixture achieved a 28-day cube compressive strength of 124.6 MPa and an apparent density of approximately 1982 kg/m^3^, realizing a compressive strength above 120 MPa at a density below 2000 kg/m^3^. The 28-day cube compressive strength of the CPM-3.0 mixture further increased to 131.7 MPa.
(2) The APM- and CPM-designed mixtures showed only slight differences in total binder content, water-to-binder ratio, and paste volume. However, the packing volume of the aggregate skeleton in the CPM-designed mixtures was approximately 3.5% lower than that in the APM-designed mixtures, indicating a denser skeleton structure. At the same steel fiber content, the LUHPC designed using CPM exhibited higher cube compressive strength, axial compressive strength, axial tensile strength, flexural strength, and shear strength than the APM-designed mixtures. This indicates that, under the present experimental conditions, CPM has better applicability for optimizing the main mechanical properties of ceramsite-sand-based LUHPC. Among these properties, the improvement in axial tensile strength did not vary monotonically with steel fiber content. The difference between the CPM- and APM-designed mixtures was the largest at a fiber content of 2.0%, indicating a favorable synergistic effect between an appropriate amount of steel fibers and the dense particle skeleton formed by CPM. The elastic modulus of the CPM-designed mixtures was also slightly higher overall, but the improvement was relatively limited. Therefore, the elastic modulus can be used as an auxiliary indicator for evaluating changes in material stiffness.
(3) Steel fiber content affected the workability, mechanical properties, elastic modulus, and shrinkage behavior of ceramsite-sand-based LUHPC. As the steel fiber content increased from 1.5% to 3.0%, the workability of LUHPC designed using both models decreased, whereas its compressive, tensile, flexural, and shear properties generally improved, and the elastic modulus increased slightly. In terms of shrinkage, steel fibers restrained the shrinkage deformation of LUHPC, but their restraining effect showed a diminishing marginal benefit with increasing fiber content. When the fiber content in the APM-designed mixtures increased from 1.5% to 3.0%, the 60-day shrinkage decreased by 14.1%. The corresponding reduction in the CPM-designed mixtures was 13.3%. At the same fiber content, the shrinkage of the CPM-designed mixtures was consistently lower than that of the APM-designed mixtures, indicating that CPM has certain advantages in shrinkage control.
(4) Considering mechanical performance, shrinkage control, workability, and material cost, a steel fiber content of approximately 2.0% is recommended as a reference range for ceramsite-sand-based LUHPC. Although further increasing the steel fiber content to 3.0% can further improve some mechanical properties, it reduces the workability of the fresh mixture and provides only limited additional shrinkage-restraining benefits. This study mainly focused on the mix design method and basic properties of ceramsite-sand-based LUHPC, while durability-related properties, such as impermeability, freeze–thaw resistance, and carbonation resistance, were not systematically investigated. Future studies may further examine the durability and service performance of ceramsite-sand-based LUHPC.

## Figures and Tables

**Table 1:** Ceramsite Sand Gradation Calculation Table.

Particle size (mm)	2.36	1.18	0.6	0.3
$p^{\prime }\left(d\right)$	1	0.605	0.280	0

**Table 2:** Particle Size and Density Parameters of Ceramsite Sand.

Particle size (mm)	0.3–0.6	0.6–1.18	1.18–2.36
Bulk density (kg/m^3^)	740.32	598.34	548.56
Apparent density (kg/m^3^)	1875	1685	1500

**Table 3:** Experimental Mix Proportions (kg/m^3^).

No.	0.3–0.6	Aggregate 0.6–1.18	1.18–2.36	Cement	Fly Ash	Silica Fume	Water	Superplasticizer	Steel Fiber
APM-1.5	184	192	208	637	209	149	179	24.8	117.7
APM-2	184	192	208	637	209	149	179	24.8	157
APM-3	184	192	208	637	209	149	179	24.8	235.5
CPM-1.5	294	118	176	628	179	195	180	25	117.7
CPM-2	294	118	176	628	179	195	180	25	157
CPM-3	294	118	176	628	179	195	180	25	235.5

Note: APM and CPM refer to the mix proportions designed using the Andreasen packing model and the compressible packing model, respectively. The number following “-” represents the fiber content.

**Table 4:** Standard Values and Strength Grades of Cube Compressive Strength.

No.	Strength Grade	Compressive Strength Standard Value (MPa)	Average Value (MPa)	Coefficient of Variation
APM-1.5	LC100	105.4262	111.3	0.032082
APM-2	LC110	110.9685	115.7	0.02486
APM-3	LC120	124.0269	125.4	0.006656
CPM-1.5	LC110	114.4323	117.1	0.013849
CPM-2	LC120	120.258	124.6	0.021184
CPM-3	LC120	129.3314	131.7	0.010933

## Data Availability

The original contributions presented in this study are included in the article. Further inquiries can be directed to the corresponding author.
